# Strategies to Achieve High Strength and Ductility of Pulsed Electrodeposited Nanocrystalline Co-Cu by Tuning the Deposition Parameters

**DOI:** 10.3390/molecules25215194

**Published:** 2020-11-08

**Authors:** Killang Pratama, Christian Motz

**Affiliations:** 1 Chair of Materials Science and Methods, Saarland University, 66123 Saarbrücken, Germany; motz@matsci.uni-sb.de; 2 Department of Metallurgical Engineering, Institute Technology of Bandung, Bandung 40132, Indonesia

**Keywords:** nanocrystalline, pulsed electrodeposition, high tensile ductility, copper, cobalt

## Abstract

Strategies to improve tensile strength and ductility of pulsed electrodeposited nanocrystalline Co-Cu were investigated. Parameters of deposition, which are pulse current density, duty cycle, and pulse-on time were adjusted to produce nanocrystalline Co-Cu deposits with different microstructures and morphologies. The most significant improvement of strength and ductility was observed at nanocrystalline Co-Cu deposited, at a low duty cycle (10%) and a low pulse-on time (0.3 ms), with a high pulse current density (1000 A/m^2^). Enhancement of ductility of nanocrystalline Co-Cu was also obtained through annealing at 200 °C, while annealing at 300 °C leads to strengthening of materials with reduction of ductility. In the as deposited state, tensile strength and ductility of nanocrystalline Co-Cu is strongly influenced by several factors such as concentration of Cu, grain size, and processing flaws (e.g., crystal growth border, porosity, and internal stresses), which can be controlled by adjusting the parameters of deposition. In addition, the presence of various microstructural features (e.g., spinodal and phase decomposition), as well as recovery processes induced by annealing treatments, also have a significant contribution to the tensile strength and ductility.

## 1. Introduction

Nanocrystalline Co is a high strength material with outstanding physical properties; however, this type of material exhibits low ductility with a typical elongation at a fracture below 10%, which is a common problem in nanocrystalline materials [1,2,3]. Low ductility in nanocrystalline materials could be influenced by several factors, such as formation of processing flaws (e.g., porosity), low plastic strain capability, and early strain localization and failure [4]. In nanocrystalline cobalt, alloying with other elements, e.g., Co-Cu alloys, is expected to enhance ductility caused by formation of the modified microstructures which change plasticity and deformation behavior. However, previous work on bulk high pressure torsion (HPT)-processed Cu-rich nanocrystalline Co-Cu [5] showed that an elongation at fracture of less than 10% was still reported. In addition, observation of micro-mechanical properties of the pulsed electrodeposited (PED) Co-rich nanocrystalline Co-Cu still reported unclear information [6]. Therefore, a study on mechanical properties and improvement in ductility of nanocrystalline Co-Cu needs further investigation.

Improving ductility of nanocrystalline materials, including nanocrystalline Co-Cu alloys, can be performed by controlling the microstructure and the formation of processing flaws [7,8,9,10]. The routes and parameters of materials processing will be key roles here. Currently, there are three possible methods to produce bulk tensile specimen of nanocrystalline materials, which are electrodeposition (ED) [1,2,11,12], severe plastic deformation (SPD) [5,13,14], and mechanical alloying and compaction (MA) [15,16]. In this paper, synthesis of bulk nanocrystalline Co-Cu through the pulsed electrodeposition (PED) will be conducted due to a simple route of production and a wide range of controlling process parameters. Of course, different characteristic and a number of lattice defects (e.g., dislocation, voids, and twins) could be also produced from different processing routes, but it is not studied here. In the PED-processed nanocrystalline materials, adjusting deposition parameters (for instance: current density, pulse-on time, and duty cycle) has a significant influence on the resulting microstructure as well as on the porosity, which has a direct impact on their mechanical properties [11,12,17,18,19]. It is also possible to improve the mechanical properties of nanocrystalline materials, particularly nanocrystalline Co-Cu, through specific annealing treatment that leads to microstructural changes (e.g., spinodal and phase decomposition) and recovery processes (e.g., relaxation of grain boundaries and internal stresses) [6,20,21]. Improvement in strength and ductility of PED-processed nanocrystalline Co-Cu is expected by tuning deposition parameters as well as annealing treatment.

In the present work, the influences of the deposition parameters (e.g., pulse current density, duty cycle, and pulse-on time) on the microstructure and chemical composition, distribution, as well as formation of processing flaws, is investigated. The effect of annealing treatments on tensile strength and ductility is also studied by discussing the possible microstructural changes and recovery processes (i.e., release of internal stresses and absorbed hydrogen). This work addresses preferred deposition parameters as well as annealing treatment conditions to produce high strength and ductility of nanocrystalline Co-Cu.

## 2. Results and Discussion

### 2.1. Effect of Deposition Parameters on the Properties of Deposits

**Figure 1:**
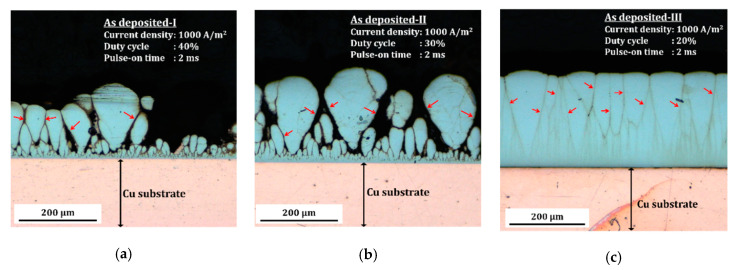
Cross-sectional images of nanocrystalline Co-Cu deposited at various duty cycle of (**a**) 40%, (**b**) 30%, and (**c**) 20% at a pulse current density of 1000 A/m^2^ and a pulse-on time of 2 ms. The films were deposited at the same number of pulse cycles for different time of deposition which are (**a**) 13 h, (**b**) 8.5 h, and (**c**) 6.5 h to obtain the 300 µm of thicknesses (current efficiency 80–90%).

Extensive studies and investigations were carried out to find specific ranges of deposition parameters of nanocrystalline Co-Cu. According to previous work [6,22], deposition at a pulse current density of 500 A/m^2^ leads to the formation of deposits with inhomogeneous distribution of microstructure and chemical composition, while porous and unstable (powdery and with high internal stresses) deposits were produced at a pulse current density of 1500 A/m^2^. Compact and homogeneous nanocrystalline Co-Cu deposits could be obtained at pulse current densities of 750–1000 A/m^2^; thus, this range was selected for the deposition of bulk nanocrystalline Co-Cu. Subsequently, these investigations were continued to find a specific range of working duty cycles and pulse-on times. Figure 1 shows cross-sectional images of nanocrystalline Co-Cu films deposited at various duty cycles at the same pulse current density (1000 A/m^2^) and pulse-on time (2 ms). According to the theory proposed by Fischer [23], deposited Co-Cu films show a combination of field-oriented texture (FT) and unoriented dispersion (UD) growth types with a clear observation of crystal growth borders (marked with red arrows in Figure 1). Figure 1a,b show that deposition at the duty cycles of 30% and 40% produce porous and unstable deposits. On the other hand, a relatively compact deposit up to ~300 µm of thicknesses is obtained in the sample deposited at a duty cycle of 20% (see Figure 1c). However, homogeneous and stable deposits of nanocrystalline Co-Cu are only visible up to ~400 µm of thicknesses at a pulse-on time of 2 ms [6]. The inhomogeneity is strongly affected by significant concentration decrease of electrolyte components (e.g., Co and Cu ions and Saccharin), which leads to the formation of coarser grains with higher Cu concentration at the area near the surface of deposits [22]. Ideally, concentration of the electrolyte components must be kept at the same level, for example, by utilizing titration and electrolyte circulation to produce thick (>1 mm) and homogeneous deposits. However, in the present work, all deposits were produced through the single stage of deposition with no addition of electrolyte components during deposition. Further investigations reported that homogeneous and stable deposits up to ~700 µm of thicknesses could be obtained through the single stage of deposition at the pulse-on times of 0.3 ms–0.5 ms (duty cycle of 20%). Deposition at pulse-on times lower than 0.3 ms is not possible due to formation of powdery deposits. In summary, high-quality nanocrystalline Co-Cu films could be deposited at a specific range of deposition parameters: (i) Pulse current densities of 750 A/m^2^–1000 A/m^2^, (ii) pulse-on times of 0.3 ms–0.5 ms, and (iii) duty cycles of 10–20%.

**Figure 2:**
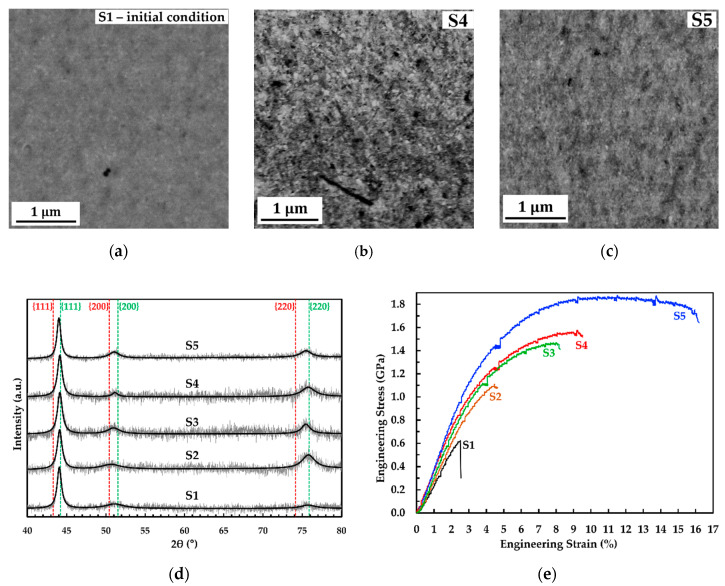
Back-scattered electron (BSE) image of initial microstructure of (**a**) S1, (**b**) S4, (**c**) S5 in planar mode; (**d**) X-ray diffraction (XRD) patterns of S1–S5. The red and green vertical dashed lines indicate the positions of the face-centered cubic (fcc) phase lines of Cu and Co, respectively. The continuous black thick lines correspond to the fits for the measured XRD patterns; (**e**) Engineering stress vs. strain curves of static tensile tests of samples S1–S5 at a strain rate of 1.0 × 10^−3^ s^−1^. Detailed descriptions for each sample are shown in Table 2.

In the present work, the deposition parameters were varied, i.e., current density, duty cycle, and pulse-on time, as described in Table 1. Microstructural investigation through scanning electron microscopy (SEM) and X-ray diffraction (XRD) measurements were performed to investigate the impact of deposition parameters. Back-scattered electron (BSE) images of initial microstructure of sample 1 (S1) (Figure 2a) and sample 5 (S5) (Figure 2c) reveal that homogeneous and pure nanocrystalline structure with grain sizes of less than 100 nm are observed, whereas S2 and S3 exhibit the identical microstructure (BSE images are not shown here). On the other hand, a BSE image of initial microstructure of S4 (Figure 2b) shows slightly coarser grains compared to S1 and S5, which is strongly influenced by the reduction of the pulse current density. XRD patterns of S1–S5 (see Figure 2d) show no clear differences of phase or crystal orientation, whereas identical XRD patterns with a strong (111) peak are detected in S1–S5. However, the different shape of XRD-peaks (e.g., peak intensity and peak broadening) can be taken into consideration as different grain size and microstrain effects (e.g., internal stresses). Caused by limited resolution of SEM–BSE images, grain size measurements were carried out by analyzing the XRD lines of individual samples through full width at half maximum (FWHM) method of the Scherer and Williamson–Hall approaches. The Scherer approach shows that S1–S5 have identical grain at the range of 7–12 nm. On the other hand, the Williamson–Hall approach shows more significant differences of grain size mainly for S4, which conforms more to the microstructure SEM image. Of course, a more valid grain size number can be obtained through transmission electron microscopy (TEM), but it is not conducted here. According to previous work [6], the grain size number from TEM images (~23 nm) is between the grain size number from Scherer (~14 nm) and Williamson–Hall (~31 nm) approaches. According to the previous measurement characteristic, S4 may exhibit grain size in the range of 9–72 nm, which is the biggest among S1–S5. The energy dispersive X-ray spectroscopy (EDS) measurement shows that the S1 consists the lowest Cu content, while the S4 and S5 have the highest Cu-content. In comparison with S1, higher Cu-content in S4 and S5 may be caused by a deposition at a lower current density and a lower duty cycle, respectively. The possible effect of grain size and Cu-concentration on strength and ductility will be discussed further in Section 2.2.

### 2.2. Effect of Deposition Parameters on the Mechanical Properties

**Figure 3:**
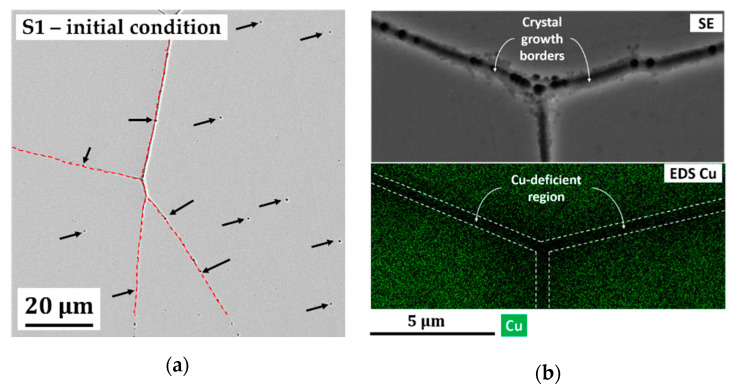
(**a**) Secondary electron (SE) image of initial condition of tensile specimen of S1 surface (planar mode), which shows the presence of micropores (marked with black arrows) and crystal growth borders (marked with red dashed line); (**b**) combined high magnification of a secondary electron (SE) image (upper section) and energy dispersive X-ray spectroscopy (EDS) elemental map of Cu (lower section) shows a Cu-deficient region along crystal growth borders (planar mode).

In this section, the effect of deposition parameters on the mechanical properties of individual samples is discussed. The stress–strain curve and the detailed data of S1–S5 are available in Figure 2e and Table 2. Figure 2e depicts an engineering stress vs. strain curve of a static tensile test of S1, which was deposited at the highest pulsed current density, duty cycle, and pulse-on time. According to the curve, S1 possess a brittle behavior in which a failure occurs even in the elastic regime (fracture stress and strain are 0.62 GPa and 2.54%, respectively). Previous work on the tensile test of bulk HPT-processed Cu-rich nanocrystalline Co-Cu [4] showed that a minimum yield strength of 0.8 GPa could be achieved and it should be higher for the Co-rich systems. A secondary electron (SE) image of the initial surface condition of S1 (Figure 3a) shows the presence of microporosity with sizes of 0.5–1.0 µm (marked with black arrows) and crystal growth borders with an average distance of 84.14 ± 44.34 µm (marked with red dashed line), which could be possible spots for crack initiation. In addition, a combined SE-EDS image (Figure 3b) reports a local Cu-deficient region along the crystal growth borders. It is believed that these crystal growth borders are more brittle compared with the surrounding regions caused by lower Cu, as well as higher Co concentration, and it is strongly supposed that the crack initiation started here.

**Figure 4:**
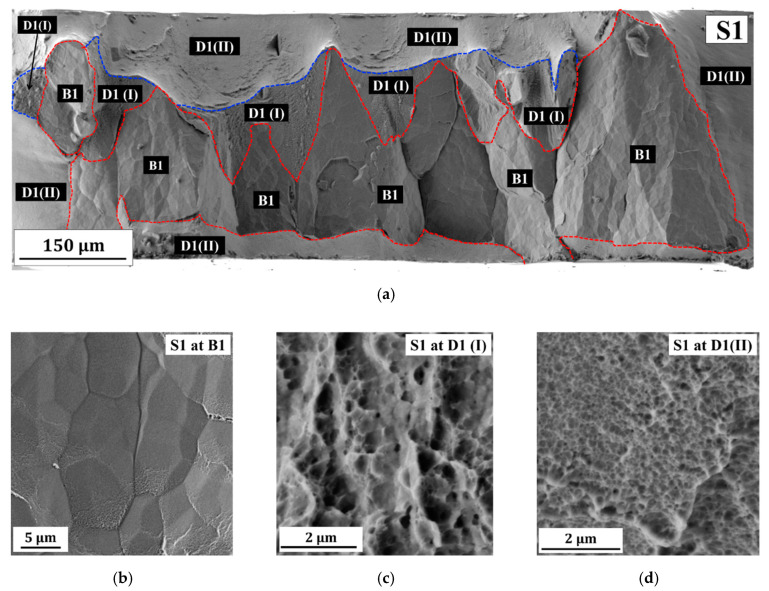
(**a**) A Secondary electron (SE) image of fracture surface of sample 1 (S1) shows brittle (B1) and ductile (D1(I) and D1(II)) fracture regions; (**b**–**d**) SE images of detailed fracture surface of S1 at three different regions marked at (**a**), which are (**b**) brittle (B1) and (**c**,**d**) ductile (D1(I), and D1(II)) fracture regions.

**Figure 5:**
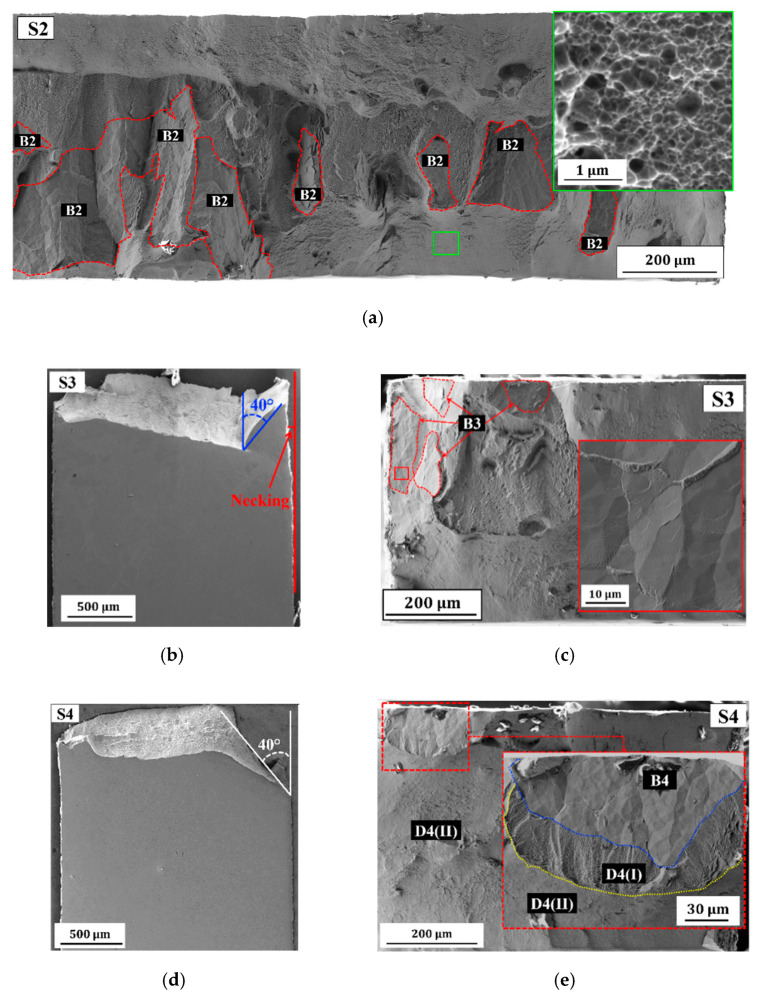
(**a**) A SE electron image of the fracture surface of sample 2 (S2) showing brittle (B2) and ductile (rest) fracture regions (inset shows a detailed image of the ductile fracture surface taken at the area marked with the green box). (**b**,**d**) SE images of surface morphology at gauge section after tensile test of (**b**) sample 3 (S3) and (**d**) sample 4 (S4); (**c**) a SE image of the fracture surface of S3 showing the brittle (B3) and ductile (rest) fracture regions (inset show a detailed image of the brittle region marked with the red box); (**e**) a SE image of the fracture surface of S4 showing brittle (B4) and ductile (D4(I) and D4(II)) fracture regions.

Figure 4a shows that the fracture surface of S1 is dominated by brittle fracture (region B1) and a clear fracture of the crystal growth borders is confirmed by detailed observation of B1 (Figure 4a,b). Other factors, such as overall low Cu concentration, impurities, and processing flaws caused by deposition at high pulse current density, could also contribute to the brittle failure of the crystal growth borders. Interestingly, a moderate fraction of ductile fracture regions (see Figure 4a) and micro-dimple structures are observed within the brittle fracture region (see Figure 4c,d). It is believed that the PED-processed nanocrystalline Co-Cu exhibits ductile behavior in the absence of these processing flaws. Hence, an improvement in ductility can be achieved by reducing the number of processing flaws, for instance, through adjustment of deposition parameters (e.g., reducing duty cycle). For example, sample 2 (S2), which was deposited at a lower duty cycle shows a slightly improved ductility (see Table 2 and Figure 2b) with no distinct porosity and crystal growth borders observed. A fracture image of S2 (Figure 5a) shows that the fraction of the brittle fracture regions (B2) is slightly lower compared to S1, and the fracture surface is dominated by a ductile fracture with various dimple sizes. In addition, the EDS measurement shows that S2 exhibits a higher Cu content than S1, which might also contribute to the improvement in ductility (see Table 1). However, ductility of S2 is still low, with a total elongation at the fracture remaining below 10%, and it needs further improvement.

Sample 3 (S3) and sample 4 (S4) were deposited at lower pulse-on time and pulse current density compared with S2, respectively. EDS measurements show that S3 and S4 have a higher Cu concentration compared to S1 and S2 (see Table 1). In comparison with S1 and S2, a significant improved ductility is observed in which elongation at fracture of almost 10% is achieved (see Table 2 and Figure 2b). Surface morphology images at the gauge section of S3 (Figure 5b) and S4 (Figure 5c) exhibit a shear fracture at 40° angle relative to the tensile load direction as an indication of ductile fracture, and also some small amount of necking as an evidence of plasticity prior to ductile fracture. Fracture surface images of S3 (Figure 5d) and S4 (Figure 5e) confirm a significant reduction of brittle fracture regions (B3 and B4) compared with S1 and S2, whereas here this type of fracture is only observed in some spots.

**Figure 6:**
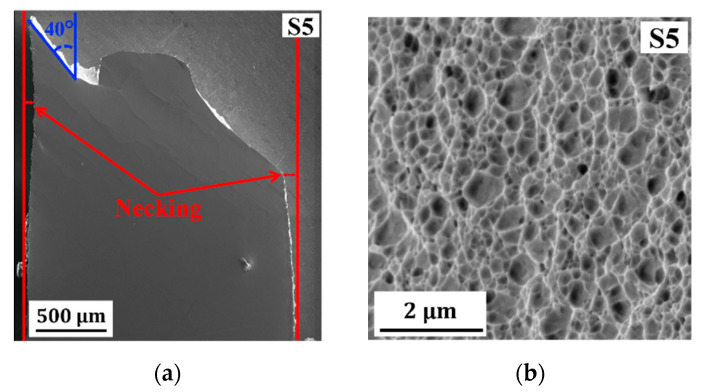
(**a**) SE images of surface morphology at the gauge section after tensile test of sample 5 (S5); (**b**) a SE image of the fracture surface of sample 5 (S5) showing ductile fracture with dimple structures.

Sample 5 (S5) was deposited at the lowest duty cycle (10%) and pulse-on time (0.3 ms). EDS measurement confirms that S5 has the highest Cu content among S1–S5 (see Table 1). An engineering stress vs. strain curve of S5 (Figure 2b) shows that a high tensile strength and ductility with a total elongation at fracture of 16.20% is observed (see the data in Table 2). A surface morphology image at the gauge section of S5 (Figure 6a) shows a moderate necking with a shear fracture at 40° angle relative to the tensile loading direction as found also in S3 and S4. No brittle fracture regions are found in S5 in which ductile fracture with dimple structures is observed (Figure 6b). Deposition at a duty cycle of less than 10% is not preferred considering the required processing time. Moreover, it also leads to deposits with higher Cu concentration and coarser grains, which results in a reduction of strength with an increased ductility.

The ductility of materials is controlled by factors such as sample geometry (e.g., specimen thickness and length, notches, etc.), chemical composition, microstructure (e.g., grains and crystal orientation), and processing flaws (e.g., porosity and internal stresses). Of course, lattice defects (e.g., dislocations) and their mobility have also a high contribution to ductility, but this is not discussed in this paper. The geometry of the tensile specimens, for instance, gauge thickness and length, is important to accommodate the plastic elongation, which contributes on ductility. According to some standards for flat tensile test specimens [24,25], the equation of $L_{o}=5.65\;\sqrt{S_{o}}$ is commonly used to calculate the proportional dimension of gauge length (L_o_) from the cross-sectional area of gauge section (S_o_). In the present work, the width (2 mm) and length (6 mm) of the gauge section are kept constant, while the specimen thicknesses (0.31–0.54 mm) are not intentionally varied due to sample preparations (grinding and polishing). According to the equation and cross-sectional data, the applied gauge length (6 mm) complies the minimum requirement of gauge lengths of 4.5–5.9 mm; thus, the gauge length should have no influence. Considering the thickness effect, some works [26,27,28] reported that the major effect of specimen thickness is only observed at the non-uniform plastic elongation (post-necking zone), which is not observed in samples S1–S4. Of course, the non-uniform plastic elongation and highest elongation at fracture are observed in S5, but this sample has the smallest sample thickness among samples S1–S5, which does not comply with the thickness effect theory. Thus, it is believed that sample thickness has no significant effect here.

Considering no significant correlation between ductility with specimen thickness, it is believed that an improved ductility is influenced by other factors. Dislocation mobility and plastic deformation capability are key roles to the ductility of materials. These factors are strongly influenced by internal properties of materials, such as chemical composition, grain size, and presence of processing flaws. Possible contributing factors, such as Cu concentration, grain size, crystal growth borders, porosity, and internal stresses are discussed with respect to the deposition parameters.

The first factor is related to Cu concentration. Pure nanocrystalline Co dominantly exhibits a hexagonal closed pack (hcp) structure, which is known to be brittle due to a limited number of active slip systems. The addition of Cu in the nanocrystalline Co is addressed to change the hcp- to fcc-structure, which exhibit a higher number of active slip systems. In addition, some research in pure nanocrystalline Cu [29,30] reported good ductility for more than 10% of elongation at fracture; thus, improvement of ductility is expected in nanocrystalline Co-Cu. According to thermodynamic data, deposition of Cu is easier than Co caused by a more positive standard reduction potential ($E_{{\mathrm{Cu}}^{2+}/\mathrm{Cu}}^{0}$ = +0.34 V vs. SHE and $E_{{\mathrm{Co}}^{2+}/\mathrm{Co}}^{0}$ = −0.28 V vs. SHE). The increase of Cu concentration in S3 and S4 is affected by this effect due to deposition at shorter pulse-on time (S3), and lower pulse current density (S4) compared to S1, respectively. Higher Cu content is also obtained at the samples deposited at longer off-time, such as observed in S2 and S5 (compared with S1), which is caused by galvanic reaction of deposited Co with Cu ions in the electrolyte when the pulsed current is off. The other possible effect relating to the presence of Cu is the formation of Co-Cu/Cu multilayered structure sizes of 1–3 nm prior to galvanic reaction when the pulsed current is off [31]. This nano-scale multilayered structure may have an impact on the ductility of the nanocrystalline Co-Cu. In the previous work [6], investigation on the Co and Cu atomic distribution was conducted through the atom probe tomography (APT) measurement in the identical sample. However, the formation of Co-Cu/Cu multilayers and Co- or Cu-segregations in the nanometer scale cannot be detected from the APT measurement. Further observation through the higher resolution of APT or TEM is needed to investigate the possible formation of these multilayers structure. In the nanocrystalline Co-Cu, ductility increases with increasing Cu content, but it should be considered that high Cu content may lead on the decrease of strength. There must be an optimum Cu content to achieve high tensile strength and good ductility.

The second factor is related to the grain size. According to the XRD grain size analysis, S4 and S5 exhibit bigger grain size compared with S1–S3 (see Table 1). Although the grain size is not accurately measured here (only estimation of grain size range), distinct observation of coarser grains in S4 is real from a BSE image (Figure 2b). In comparison with S1 and S2, more ductility can be obtained from S4 and S5. It is believed that coarser grain (i.e., still in the nanocrystalline range) have a contribution on the increase of ductility. According to Meyers [4], low plasticity in the nanocrystalline materials is contributed by a significantly lower number and mobility of dislocations within individual grain. The number and mobility of dislocations within individual grain increase with increasing grain size; thus, more plastic deformation can be obtained (i.e., more ductility). Deposition at lower current density (S4) and lower duty cycle (S5) produces the nanocrystalline Co-Cu with higher Cu-content and coarser grain. The influence of higher Cu-content on the increase of grain size in the nanocrystalline Co-Cu has been observed by Müller [22].

The third factor is related to the formation of crystal growth borders. The crystal growth was introduced by Fischer [23], relating to the nucleation and growth mechanism in the electrochemical deposition. In the nanocrystalline materials, one “crystal” growth may consist up to hundreds or thousands of nano-grains, which may have similar crystal orientations (fiber texture). Usually, many “crystals” are growing at the same time and will get into contact, at some point, to form crystal growth borders. At these borders, the chemical composition may vary (depletion of alloying element in the electrolyte, agglomeration of impurities, etc.). In the present work, inhomogeneous concentration of Co and Cu is observed at crystal growth borders in S1, as shown in Figure 3b. The formation of these borders at the surface of other tensile specimens (S2–S4) is not observed. Of course, these borders are believed remaining in the samples, and the fracture surface images of S2–S4 (see Figure 5a,c,e) show that the fracture of crystal growth borders can be reduced significantly with improved ductility. These borders are not even found in the fracture surface of S5. Adjusting deposition parameters (i.e., longer off time, lower current density, etc.) has a significant impact to reduce the formation of these borders through different mechanisms.

The fourth factor is related to hydrogen evolution and formation of porosity. Hydrogen evolution at the cathode leads on the reduction of current efficiency and the formation of porosity due to trapped hydrogen gas, and it cannot be avoided during deposition at high current density. According to works by some authors [12,32,33], the observance of strong intensity (111) orientation in samples S1–S5 (see Figure 1a), which reflects inhibited lateral growth mode, is an indication of the hydrogen gas presence during deposition. Adjustment of parameters of deposition, such as reducing duty cycle (S2–S5), shortening pulse-on time (S3 and S5), and lowering pulse current density (S4) are intended to reduce the hydrogen evolution, which has a direct impact on number of porosities, such as discussed in literatures [17,34]. Of course, it is believed that some porosities are still in the deposits (samples S2–S5), but microstructure investigation confirms that no distinct porous formation is observed in samples S2–S5. This non-observed pore formation is, maybe, related to the smaller size of porosities compared with S1, which influences ductility. Further investigations on the critical size and number of pores should be conducted in the future to obtain a more comprehensive understanding between parameters of deposition, porosity, and ductility.

The last factor is related to internal stresses. The slightly different XRD peak form (e.g., peak intensity and peak broadening) of individual crystal textures in samples S1–S5 (see Figure 1a) is an indication of different concentration of micro-strain in the different nanocrystalline Co-Cu deposits (see Table 1). Internal micro-stress is one of contributing factor to the micro-strain broadening. During the pulsed electrodeposition process, stress generation, and relaxation occur during pulse current-on and pulse current-off, respectively [35,36,37]. The internal stresses may have an influence on a brittle behavior in deposited materials. Reducing duty cycle (S2–S5) and shortening pulse-time (S3 and S5) are purposed to extend the time for stress relaxation and shorten the time for stress generation, respectively. Thus, lower internal stresses are expected as well as an improvement of ductility. Some works by Xu [36,37] also reported the formation of twins in nanocrystalline Cu during stress relaxation when the pulse current is off, which has an impact to the strengthening of materials.

The current investigation shows that most the significant improvement on tensile strength and ductility is observed in S5, which was deposited at a combination of significantly low duty cycle (10%) and short pulse-time (0.3 ms), where no reduction in pulse current density is needed (i.e., no decrease of strength). Thus, deposition at these parameters is favored to achieve high tensile strength and good ductility of nanocrystalline Co-Cu. Surely, further investigation and optimization, such as effect of deposition temperature and electrolyte composition are required to obtain the optimum results. These results show the importance of deposition parameters in the massive improvement on ductility of nanocrystalline Co-Cu.

### 2.3. Effect of Annealing Temperatures on Ductility

**Figure 7:**
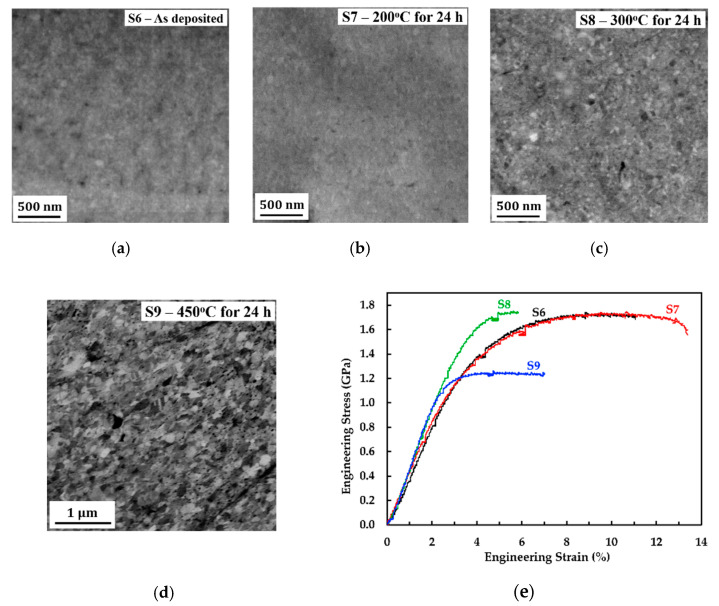
(**a**–**d**) BSE images of initial microstructure of (**a**) sample 6 (S6), (**b**) sample 7 (S7), (**c**) sample 8 (S8), and (**d**) sample 9 (S9) in planar mode; (**e**) engineering stress vs. strain curves of static tensile test at a strain rate of 1.0 × 10^−3^ s^−1^ of sample 6 (S6)–sample 9 (S9). The description for each sample is shown in the Table 3.

The effect of annealing temperatures on the ductility of Co-rich nanocrystalline Co-Cu is investigated. Figure 7a shows a back scattered electron (BSE) image of micrograph of sample 6 (S6), which was deposited at a pulse current density, duty cycle, and pulse-time of 900 A/m^2^, 10%, and 0.3 ms, respectively. A reduced pulse current density compared with S5 is intended to enhance ductility further. In comparison with S5, nanocrystalline Co-Cu with coarser grains (Figure 7a) and higher Cu content (Table 3) is observed in S6 due to deposition at lower pulse current density. Samples deposited at the same parameter with S6 were subjected to annealing treatment at 200 °C (S7), 300 °C (S8), and 450 °C (S9) for 24 h to investigate the effect of annealing on mechanical properties. BSE images (Figure 7b–d) show that no significant grain changes (e.g., grain coarsening) are detected at the sample annealed at 200 °C (S7) and 300 °C (S8), while slight grain coarsening is observed at the sample annealed at 450 °C (S9). Previous work on nanocrystalline Co-Cu (28 at.% Cu) [6] confirms identical results with these findings.

**Figure 8:**
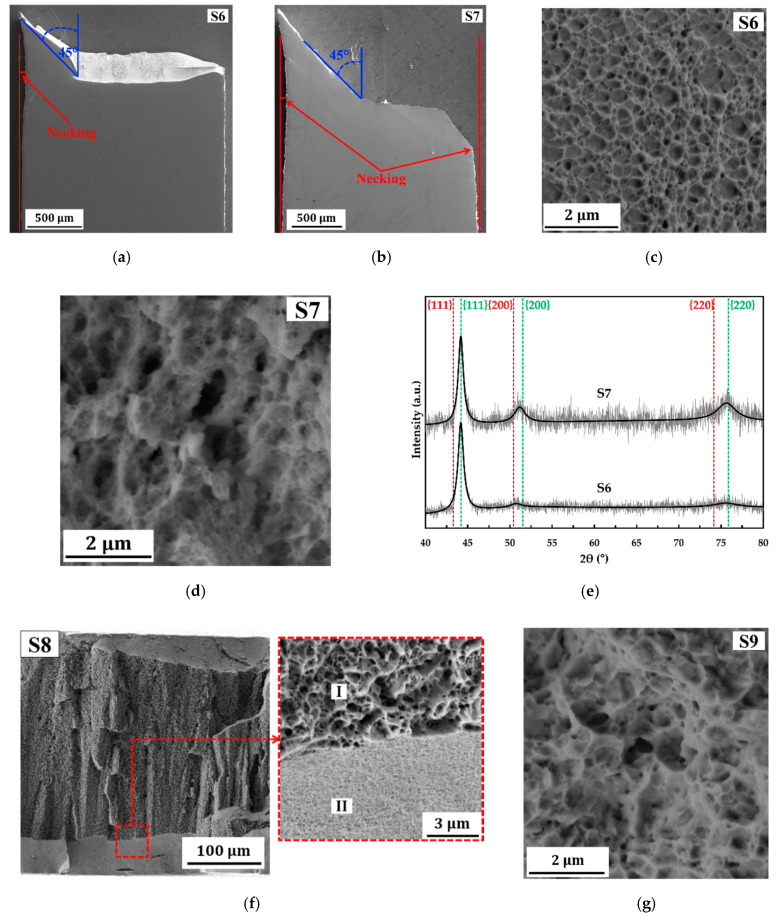
(**a**,**b**) SE images of surface morphology at the gauge section after tensile test of (**a**) sample 6 (S6) and (**b**) sample 7 (S7); SE images of fracture surface of (**c**) sample 6 (S6), (**d**) sample 7 (S7), (**f**) sample 8 (S8) and (**g**) sample 9 (S9); (**e**) XRD patterns of samples S6 and S7.

Figure 7e depicts engineering tensile stress vs. strain curves of S6–S9 at a strain rate of 1.0 × 10^−3^ s^−1^ and the detailed data are provided in Table 3. The stress vs. strain curve of S6 confirms that the as deposited state shows good ductility, with a total elongation at fracture of 11.37 ± 0.24%. In comparison with S6, annealing at 200 °C for 24 h (S7) leads on the improvement of ductility while the strength is maintained at the same level (see Figure 7e and Table 3). Surface morphology images at a gauge section of S6 (Figure 8a) and S7 (Figure 8b) show moderate necking with shear fracture at an angle of 45° relative to the tensile load direction as indications of ductile fracture. Fractographies of S6 (Figure 8c) and S7 (Figure 8d) also show ductile fracture surfaces with a dimple structure. In comparison with S5, a decrease of ductility and strength are confirmed in S6, although the pulse current density has been lowered. It is believed that the decrease of ductility in S6 compared with S5 is caused by the thickness effect, while the decrease of strength is caused by the grain size effect. On the other hand, the improved ductility in samples S7 (compared with S6) may be caused by recovery processes, such as relaxation of internal stresses, and grain boundaries, as well as the release of absorbed hydrogen. XRD patterns of samples S6 and S7 (see Figure 8e) confirm significantly dissimilar peak intensity and peak broadening of (200) and (220) peaks indicating different concentration of micro-strains (e.g., internal stresses). Of course, better XRD resolution and other contributing measurements (e.g., absorbed hydrogen measurement) are needed to obtain exact value of those mentioned factors. However, these results could be an initial indication that the improved ductility in S7 may be caused by the recovery processes. At this point, structural transformation at 200 °C must be further investigated to reveal the influencing factors on the improvement of ductility.

In comparison with the as deposited state (S6), annealing treatment at 300 °C for 24 h (S8) leads to a strengthening of nanocrystalline Co-Cu in which the observed yield strength increases to 1.4 GPa (see Figure 7e and Table 3). The XRD measurement shows that the as deposited state nanocrystalline Co-Cu (S6) exhibits a supersaturated solid solution phase with strong (111) orientation (see Figure 8e). According to previous work [6], the nano scale spinodal or chemical decomposition of solid solution Co-Cu was detected at 300 °C, in which three regions (solid solution Co-Cu, Co-rich, and Cu-rich) were detected. The presence of Co- and Cu-rich regions with sizes of 5–10 nm, which may act as Co- or Cu-precipitates, is expected to have a direct impact to a strengthening of nanocrystalline Co-Cu (S8). However, improved ductility is not observed here with the total elongation at fracture decreases to 5.48%. The fracture image of S8 (Figure 8f) shows two ductile fracture regions with different size of dimples. Figure 8f depicts that the dimple structures with the sizes of 0.5 µm–1 µm are observed at the center of a cross-section tensile specimen (region I), while the smaller sizes of dimples are found at the area near surface (region II).

The engineering stress vs. strain curve of S9 (Figure 7e) shows that annealing at 450 °C for 24 h leads to decrease of strength and ductility in comparison with the as deposited state (S6). Interestingly, a fracture surface of S9 shows no brittle fracture surface, whereas dimple structures (Figure 8g) as indication of ductile fracture are observed. According to previous work [6], slight grain coarsening, which is also observed in S9, as well as massive phase decomposition of Co-Cu are reported after annealing at 450 °C for 24 h. It is clear enough that the reduced strength in S9 is caused by the grain coarsening. Surprisingly, phase separation of Co-Cu, which is expected to improve the mechanical stability, is unable to improve (or even maintain) the ductility of nanocrystalline Co-Cu. According to previous work [6], phase decomposition of the fcc-structured solid solution nanocrystalline Co-Cu at 450 °C for 24 h led to the formation of fcc-Cu, fcc-Co, and hcp-Co phases. It is believed that a decreased ductility is caused by the presence of an hcp-Co phase, which is known to be more brittle compared with the fcc-structure. Other factors, such as impurity segregation at grain boundaries, may also have a contribution on reduction of ductility at 450 °C. At 450 °C, the diffusion of co-deposited impurities (e.g., sulfur) to the grain boundaries is possible as shown in the nanocrystalline Co [38] and Ni [39]. The segregation of sulfur at grain boundaries have an impact on grain boundary embrittlement, which leads to the reduction of ductility.

In this paper, high tensile ductility and strength of pulsed electrodeposited nanocrystalline Co-Cu can be achieved by adjusting the parameters of deposition. Moreover, annealing treatments show some interesting and surprising results in which an improvement of ductility can also be obtained through this method. In the future, fatigue behavior of PED-processed bulk nanocrystalline Co-Cu will be also studied.

## 3. Materials and Methods

Bulks nanocrystalline Co-Cu with thickness of 600–750 µm were produced through the pulsed electrodeposition technique at copper cathode plates (21 mm × 21 mm) and double plate of titanium-mesh (120 mm × 60 mm) was used as an anode. The PED process was conducted to produce nanocrystalline Co-Cu deposits (S1–S9 samples) at different parameters of deposition (pulse current density, duty cycle, and pulse-time) as shown in Table 1 and Table 2. The times for the deposition are different for individual samples, which are 35 h for S1, 40 h for S2–S3, 50 h for S4, 69 h for S5, and 72 h for S6–S9, with a current efficiency of 80–90%. The PED process was performed with 1.10 L electrolyte containing 0.40 M CoSO_4_.7H_2_O, 0.04 M CuSO_4_, 0.20 M C_4_H_4_KNaO_6_.4H_2_O, 1.00 M Na_2_SO_4_, 0.3 M H_3_BO_3_, 2.00 g/L C_7_H_5_NO_3_S (Saccharin), and 0.20 g/L C_12_H_25_NaO_4_S (sodium dodecyl sulfate) at a constant temperature of 40 °C. Some deposits (S7–S9) were subjected to isothermal annealing in vacuum at a pressure of 10^−6^ mbar at various temperatures (200–450 °C) for 24 h to investigate the effect of annealing temperatures on the mechanical properties.

**Figure 9:**
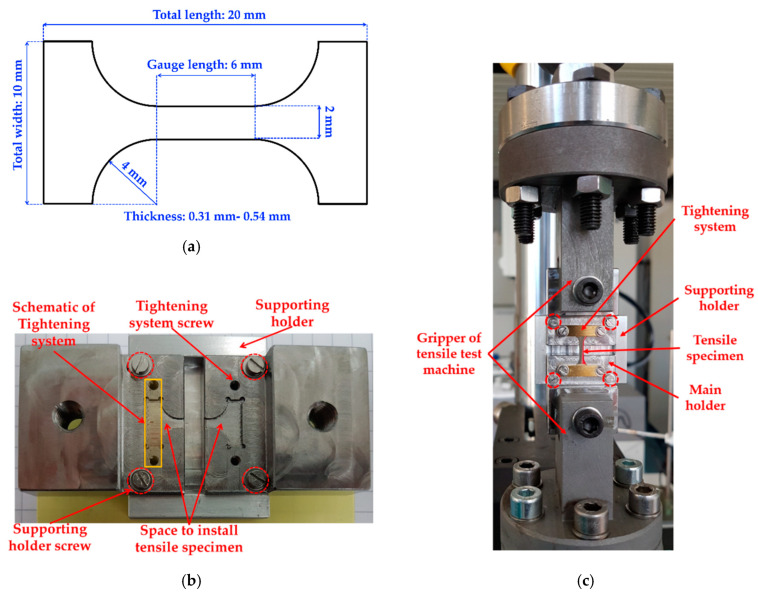
(**a**) Schematic picture of the dog-bone tensile specimen used in this work. The thickness of the specimens is intentionally varied, as shown in Table 1 and Table 3; (**b**) design of the tensile specimen holder. A supporting holder is used to support the holder and specimen just before the test, and will be removed after installation in the tensile testing machine. The red dashed circle shows some screws to remove the supporting holder. The tightening system is used to fix the tensile specimen at its place; (**c**) installation of the tensile specimen holder (including tensile specimen) in the tensile testing machine. The supporting holder must be removed after the installation (before the tensile test) by removing the screws, which are marked with red dashed circle.

All deposits were removed from copper substrate and they were subjected to cutting processes to produce two tensile specimens from each deposit. Figure 9a shows a schematic picture and dimension of a dog-bone tensile specimen used in this work. Tensile specimens were subjected to grinding and polishing procedure, and some parts (200–300 µm) of the tensile specimen thickness will be lost during this step. Polished tensile specimens were settled to the specimen holder, as shown in Figure 9b. This holder (including tensile specimen) was installed in the tensile testing machine, as demonstrated in Figure 9c. Tensile tests were carried out in a testing machine, Instron 8513 (Instron, Norwood, MA, USA), at a strain rate of 1.0 × 10^−3^ s^−1^. Caused by limitation of the samples, i.e., small dimension compared to the standard tensile specimen, the measured strain data were only recorded by the cross-head displacement of the machine with no additional devices (e.g., extensometer). Initial microstructure and fracture surface images, as well as chemical composition of tensile specimens, were investigated in the scanning electron microscope (SEM) Zeiss Sigma-VP (Jena, Thüringen, Germany) equipped with a backscattered electron (BSE) detector and energy dispersive spectroscopy (EDS) detector (Oxford Instruments, Abingdon, Oxfordshire, England) at acceleration voltages of 10–20 kV. X-ray diffraction (XRD) measurements were conducted by using Cu K-alpha radiation (λ: 1.5405980 Å) and a scan step size of 0.013°2θ/s. The XRD data were processed with Fityk software (Institute of High Pressure Physics, Warsaw, Poland) [40] for peak refinement and peak fitting. The SEM and XRD measurements were carried out at the surface of the tensile specimen next to the solution side.

## 4. Conclusions

Strategies to obtain high tensile strength and ductility of PED-processed bulk nanocrystalline Co-Cu were investigated by adjusting pulse current density, duty cycle, and pulse-time of deposition. An improved strength and ductility can be achieved through the deposition at high pulse current density (1000 A/m^2^) by reducing duty cycle and pulse-time up to 10% and 0.3 ms, respectively. Specimen deposited at this parameter shows that an elongation to fracture of 15.95 ± 0.09%, as well as yield and ultimate strength up to 1.21 ± 0.08 GPa and 1.98 ± 0.03 GPa, respectively, could be achieved. Improved ductility could also be acquired through isothermal annealing at 200 °C, whereas annealing at 300 °C leads to strengthening with reduction of ductility. It is believed that improvement on ductility and strength of PED-processed nanocrystalline Co-Cu is affected by several factors, such as Cu concentration, grain size, and number of flaws within materials (e.g., crystal growth borders, porosity, and internal stresses).

## Figures and Tables

**Table 1:** Chemical composition and grain size analysis of the tensile specimens, which were deposited at (various) pulse current density (i_p_), duty cycle (dc), and pulse-on time (t_p_). Grain size analysis is measured from the XRD lines in Figure 2a through full width at half maximum (FWHM) analysis and two different approaches: Scherer plot and Williamson–Hall plot. PED = pulsed electrodeposited.

	PED Parameters			Composition		Scherer Plot	Williamson–Hall Plot	
	i_p_ (A/m^2^)	dc (%)	t_p_ (ms)	Co (at.%)	Cu (at.%)	Grain Size (nm)	Grain Size (nm)	Microstrain (%)
S1	1000	20.0	0.5	87.1 ± 0.1	12.9 ± 0.1	7.84	12.86	1.018
S2	1000	17.5	0.5	85.6 ± 0.1	14.4 ± 0.1	7.39	7.08	0.393
S3	1000	17.5	0.3	82.8 ± 0.1	17.2 ± 0.1	8.30	7.76	0.161
S4	800	17.5	0.5	80.3 ± 0.1	19.7 ± 0.1	9.03	71.68	1.297
S5	1000	10.0	0.3	80.1 ± 0.1	19.9 ± 0.1	11.03	24.60	0.673

**Table 2:** The yield strength (σ_y_), ultimate tensile strength (σ_u_), and total elongation at fracture (ε_f_) of tensile specimens S1–S5 with different thicknesses (d) measured from the engineering stress–strain curves in Figure 2b. Two tensile specimens were mechanically tested for S2–S5.

	PED Parameters			d (mm)	Mechanical Properties		
	i_p_ (A/m^2^)	dc (%)	t_p_ (ms)		σ_y_ (GPa)	σ_u_ (GPa)	ε_f_ (%)
S1	1000	20.0	0.5	0.41	Fail at 0.62		2.54
S2	1000	17.5	0.5	0.54	0.91 ± 0.06	1.20 ± 0.03	4.57 ± 0.05
S3	1000	17.5	0.3	0.52	0.95 ± 0.05	1.51 ± 0.04	8.71 ± 0.47
S4	800	17.5	0.5	0.53	1.03 ± 0.02	1.55 ± 0.03	8.26 ± 1.26
S5	1000	10.0	0.3	0.40	1.21 ± 0.08	1.98 ± 0.03	15.95 ± 0.09

**Table 3:** Description of tensile specimens S6–S9 with the Identical thickness (D) and chemical composition which were deposited for 72 h at pulse current density (i_p_), duty cycle (DC), and pulse-on time (t_p_) of 900 A/m^2^, 10%, and 0.3 ms, respectively. Samples S7–S9 were annealed at different annealing temperatures for 24 h. The yield strength (σ_y_), ultimate tensile strength (σ_u_), and total elongation at fracture (ε_f_) are measured from the engineering stress–strain curves in Figure 7e. Two tensile specimens were mechanically tested for S6 and S9.

	d (mm)	Annealing		Composition		Mechanical Properties		
		Temp (°C)	Time (h)	Co (at.%)	Cu (at.%)	σ_y_ (GPa)	σ_u_ (GPa)	ε_f_ (%)
S6	0.31	As deposited		79.5 ± 0.0	20.5 ± 0.0	1.16 ± 0.01	1.71 ± 0.02	11.37 ± 0.24
S7	0.31	200	24	79.5 ± 0.0	20.5 ± 0.0	1.10	1.73	13.38
S8	0.31	300	24	79.8 ± 0.1	20.2 ± 0.1	1.40	1.75	5.84
S9	0.35	450	24	79.8 ± 0.1	20.2 ± 0.1	1.03 ± 0.08	1.28 ± 0.17	6.05 ± 0.97
